# Recruitment of intertidal kelps *Hedophyllum sessile* and *Alaria marginata* (*Laminariales*) to articulated and crustose coralline algal species

**DOI:** 10.1111/jpy.70024

**Published:** 2025-05-06

**Authors:** Ruby Burns, Brenton A. Twist, Patrick T. Martone

**Affiliations:** ^1^ Department of Botany and Biodiversity Research Centre University of British Columbia Vancouver British Columbia Canada

**Keywords:** calcified algae, facilitation, intertidal ecology, kelp ecology, macroalgae

## Abstract

Kelps and coralline algae are important primary producers and habitat‐builders in rocky intertidal ecosystems. On wave‐exposed shores along the west coast of North America, *Hedophyllum sessile* and *Alaria marginata* are two dominant kelp species with juveniles that often occur at a higher density on articulated corallines than other available substrates. Little is known of the mechanisms underlying this interaction. One hypothesized mechanism is that articulated coralline algae enhance kelp spore settlement and germination. This study tested this hypothesis by releasing spores from *H. sessile* and *A. marginata* onto multiple genetically identified articulated and crustose coralline species, as well as bare rock, then observing subsequent sporophyte densities. Kelp recruitment was generally higher on articulated corallines than on crustose corallines, although there was variation across coralline species. There was no significant difference between recruitment on bare rock and on articulated corallines, and recruitment was higher on bleached *Corallina vancouveriensis* than on live *C. vancouveriensis*, suggesting that this articulated coralline actively inhibits rather than promotes intertidal kelp settlement. Thus, other mechanisms, such as protection from herbivory or wave action, likely explain observed distributions of kelp recruits. This research contributes to understanding how the fine‐scale distribution of kelps is linked to that of corallines.

**Abbreviations**
ANOVA: analysis of variance
*SE*
: standard error

## INTRODUCTION

**FIGURE 1:**
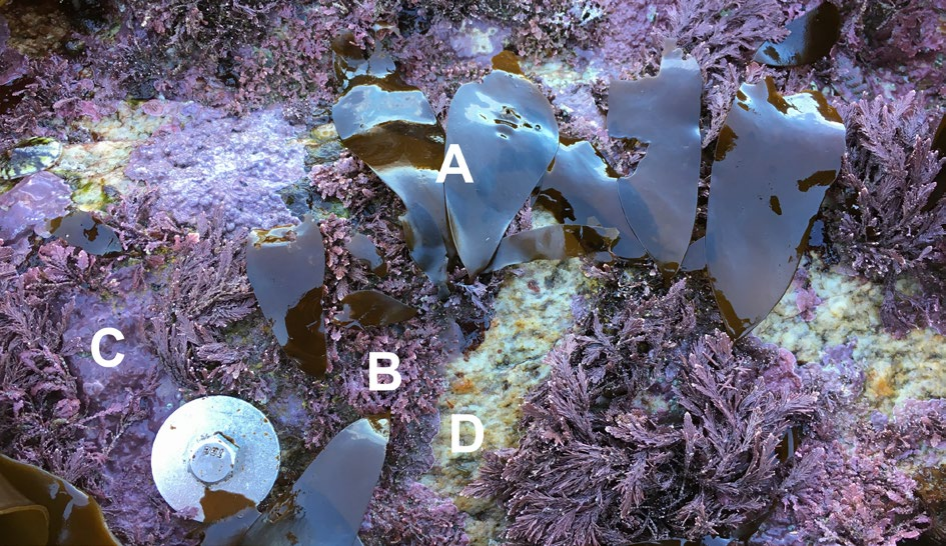
Juvenile *Hedophyllum sessile* (A) grows more frequently on the articulated coralline *Corallina vancouveriensis* (B) than crustose corallines (C) or bare rock (D). Photo taken by B.A Twist on Calvert Island, BC.

Kelps and coralline algae are two distinct groups of macroalgae that promote biodiversity in rocky intertidal ecosystems (McCoy & Kamenos, 2015; Schiel & Foster, 2015). Kelp, large brown macroalgae in the order Laminariales, is an important food source for invertebrates and provides canopy cover that protects invertebrates and understory seaweeds from desiccation during low tide (e.g., Burnaford, 2004; Lilley & Schiel, 2006; Steneck et al., 2002). Coralline algae, calcifying red algae in the orders Corallinales, Sporolithales, and Hapalidiales, create spatially complex habitat and can facilitate invertebrate settlement (e.g., Daume et al., 1999; Kelaher et al., 2001; Twist et al., 2023). Articulated corallines, specifically, appear to participate in a mutualistic relationship with kelp: Kelps shade the corallines and protect them from bleaching (Figueiredo et al., 2000; Irving et al., 2004) while corallines form a substrate that fosters high juvenile kelp recruitment compared to other substrates (Barner et al., 2016; Twist et al., 2023). In particular, juveniles of the kelps *Hedophyllum sessile* and *Alaria marginata* are observed more often on articulated coralline algal turfs, primarily composed of *Corallina vancouveriensis* (Figure 1, P.T. Martone and B.A. Twist, personal observation), than on other available substrates in the intertidal zone (Barner et al., 2016; McConnico & Foster, 2005; Milligan & DeWreede, 2000). Although this interaction between kelp and articulated corallines has been observed across multiple species and habitats, little is known about its drivers.

Corallines may influence the local distributions of kelps through intrinsic physical, chemical, and biological processes as well as the alteration of extrinsic environmental factors. These processes can affect different stages of the kelp life cycle and occur across different spatial scales, from initial spore settlement to the dislodgement of adults (e.g., Milligan & DeWreede, 2000; Parada et al., 2017). In this paper, we have focused on kelp recruitment, defined as the creation of young sporophytes (<1 mm) after spore inoculation. This is a combined measure of settlement, gametophyte survival, gametophyte reproduction, and early sporophyte survival. It has often been suggested that articulated corallines facilitate kelp recruitment by forming spatially complex turfs that provide epiphytes with greater protection from waves, refuge from herbivores, and desiccation resistance compared to crustose corallines or bare rock (McConnico & Foster, 2005; Milligan & DeWreede, 2000; Padilla, 1984; Vadas et al., 1992). Whether articulated corallines intrinsically increase kelp recruitment in isolation from these environmental factors remains an open question.

Differences in kelp recruitment among coralline species have been documented in subtidal kelps (Twist et al., 2023), but little is known about species‐specific interactions between intertidal kelps and articulated corallines. Most field observations describing intertidal kelp recruitment patterns have not identified articulated corallines beyond their morphological group (McConnico & Foster, 2005; Milligan & DeWreede, 2000). Yet even corallines within the same morphological group are known to have different physiologies; for example, the articulated coralline *Calliarthron tuberculosum* is less desiccation‐ and temperature‐resistant but more herbivore‐resistant than the articulated coralline *Corallina vancouveriensis* (Guenther et al., 2022; Padilla, 1984), and the crustose coralline *Chamberlainium tumidum* is more desiccation‐tolerant than crustose corallines in the *Lithophyllum* genus (Dethier, 1994). These physiological differences may translate to intrinsic impacts on kelp recruitment through potential differences in traits like texture, cell shedding, and chemical cues. To understand how coralline species identity influences intertidal kelp recruitment success, it is important to test multiple species with crustose and articulated morphologies.

We investigated whether the field‐observed recruitment patterns of *Alaria marginata* and *Hedophyllum sessile* on articulated corallines could be explained by differences in sporophyte recruitment in the lab. After exposing fragments of bare rock, three articulated coralline species, and three crustose coralline species to *H. sessile* or *A. marginata* spores, we then measured resulting juvenile sporophyte density on each fragment. We hypothesized that sporophyte density would be highest on articulated coralline species and lowest on crustose coralline species with intermediate density on rock. We also hypothesized that sporophyte density would be highest on *Corallina vancouveriensis* due to the observed field recruitment patterns. This study helps uncover the importance of substrate on kelp recruitment patterns and further describes the intricate relationships between coralline algae and kelp.

## MATERIALS AND METHODS

### Coralline collection

Coralline algae and bare rock were collected from multiple coastal locations around British Columbia (BC), Canada, between 2021 and 2022 (Figure S1; Table S1). The samples were collected during low tide, 0–1.5 m above the lowest astronomical tide line. For the three crustose corallines (*Crusticorallina* spp., *Lithophyllum* spp., and *Chamberlainium tumidum*) ~2 cm wide fragments were collected using a hammer and chisel. Fronds of three articulated coralline species (*Calliarthron tuberculosum*, *Corallina chilensis*, and *Corallina vancouveriensis*) were picked directly from the substrate they were growing on. The corallines were initially identified to genus or species by trained phycologists using morphological characteristics, and identifications were later confirmed using DNA barcoding (Appendix, Table S2). In the lab, the substrates were kept in a recirculating seawater table (11 μmol photons · m^−2^ · s^−1^, 16:8 h light:dark photoperiod, 10°C, pH = 7.9, 33 salinity) and cleaned with a toothbrush once a month to remove epiphytes until the start of the experiment.

### Substrate preparation

This experiment tested kelp settlement on crustose corallines, articulated corallines, and bare rock. Immediately before the spore release, each substrate was scrubbed and rinsed in seawater to remove as many epibionts as possible. The bare rock used for *Alaria marginata* settlement was not autoclaved due to practical limitations, but the rock used for *Hedophyllum sessile* settlement was autoclaved before beginning the experiment. The autoclaved rock served as a biologically relevant control, mimicking the physical properties of available substrate in the intertidal without any competing epiphytes or microbiome. Substrate fragments were placed one to a well in six‐well plates (3.5 cm diameter Thermo Scientific™ BioLite™ Microwell Plates). Each well was randomly assigned a substrate ensuring that each plate did not have two of the same substrate. For articulated corallines, the top 2 cm of each collected frond was used so the fragment would fit in the test wells. Each substrate had nine replicates.

### Kelp collection and spore release

Sorus‐bearing *Hedophyllum sessile* blades were collected from 20 individuals at Clover Point, Victoria, BC, and *Alaria marginata* sporophylls were collected from 10 individuals at North Beach, Calvert Island, BC (Figure S1). Before releasing kelp spores, the collected fertile tissue was cleaned in a 3% iodine bath for 30 s, rinsed with sterile seawater, blotted dry, then refrigerated for 20 h at 4°C in darkness (Flavin et al., 2013).

Subsequently, all kelp tissue was submerged in 2 L of 10°C autoclaved seawater to initiate spore release. The spore density was repeatedly counted with a hemocytometer, and when live spore density exceeded 100,000 spores · mL^−1^, all kelp tissue was disposed of and a final count of spore density was recorded. The spore solution was diluted with autoclaved seawater (75 minutes at 100°C) to a final spore density of 20,000 spores · mL^−1^ to maximize sporophyte production without gametophyte overcrowding (Reed et al., 1991). Each well received 10 mL of the diluted spore solution. Additional wells with glass coverslips were used for weekly monitoring of gametophyte health and development using a compound microscope, as gametophyte progress could not be tracked non‐destructively on the opaque substrates. Immediately after adding the spore solution to each well, the well plates were covered with tinfoil to block out light and kept at 10°C, following Flavin et al. (2013). After 24 h in darkness, the tinfoil was removed, and the well plates were returned to the 10°C incubator and illuminated with ~25 μmol photon · m^−2^ · s^−1^ white light at a 12:12 light:dark daylength. The light level was chosen to reduce coralline bleaching while still encouraging kelp gametophyte reproduction.

The seawater in the wells was changed each week with 10 mL of nutrient‐enriched F/2 autoclaved seawater (Guillard, 1975) to promote kelp growth and 0.66 mg · L^−1^ germanium dioxide to discourage diatom growth. Every week, the gametophyte progress in a random three of the nine coverslips was checked using a compound microscope. Sporophytes first appeared 4 weeks after spore settlement, and counting began once sporophytes on the glass coverslips began to exceed 150 μm in size.

### Sporophyte counting

Over a 2‐ to 3‐day period, sporophytes on each substrate were counted while submerged in seawater using an Olympus SZ61 dissecting microscope at 45x magnification. To accurately represent settlement and growth on the substrates, only the sporophytes visibly growing from the substrate species were counted, disregarding any sporophytes growing from epibionts or broken edges of the substrate that were not reflective of the species in question. For most substrates, the total number of sporophytes was counted over the entire substrate surface; however, some substrates had too high a sporophyte density to manually count. For these, three haphazardly chosen regions were photographed using an Olympus DP20 microscope camera to later count sporophytes using ImageJ (Fiji v.2.3.0; Schindelin et al., 2012). To account for differences in sporophyte growth rates between substrates, sporophytes were re‐counted 1 week after the first count. The highest sporophyte count of the 2 weeks was used to calculate sporophyte density for each substrate.

Each well‐plate was photographed to determine the total surface area of each substrate using the Select and Measure functions in ImageJ. Substrate surface area was used to calculate the sporophyte density on each substrate fragment (total sporophyte count ÷ total surface area). For the substrates that had too high a density for counts to be made across the whole substrate, sporophyte density was determined in each of the three pictures, with their mean sporophyte density used as an approximation for the full substrate.

### Statistical analysis

R v. 4.1.2 (R Core Team, 2017) was used for all statistical analyses. Histogram plots of the sporophyte densities for each substrate and kelp species were used to assess normality and variation. Sporophyte density was log‐transformed to better meet the assumption of normality. However, many substrates still had different variances. Because of this, a one‐way Welch's analysis of variance (ANOVA) was used to determine any significant differences between recruitment on substrates for both *Hedophyllum sessile* and *Alaria marginata*. For significant results, the Games‐Howell post hoc test was used to determine which substrates had significantly different settlement densities while accounting for unequal variances. After half of the *Corallina vancouveriensis* fragments bleached during the *A. marginata* trial, a Welch's t‐test was used to assess the impact of *Corallina vancouveriensis* bleaching on sporophyte density.

## RESULTS

**FIGURE 2:**
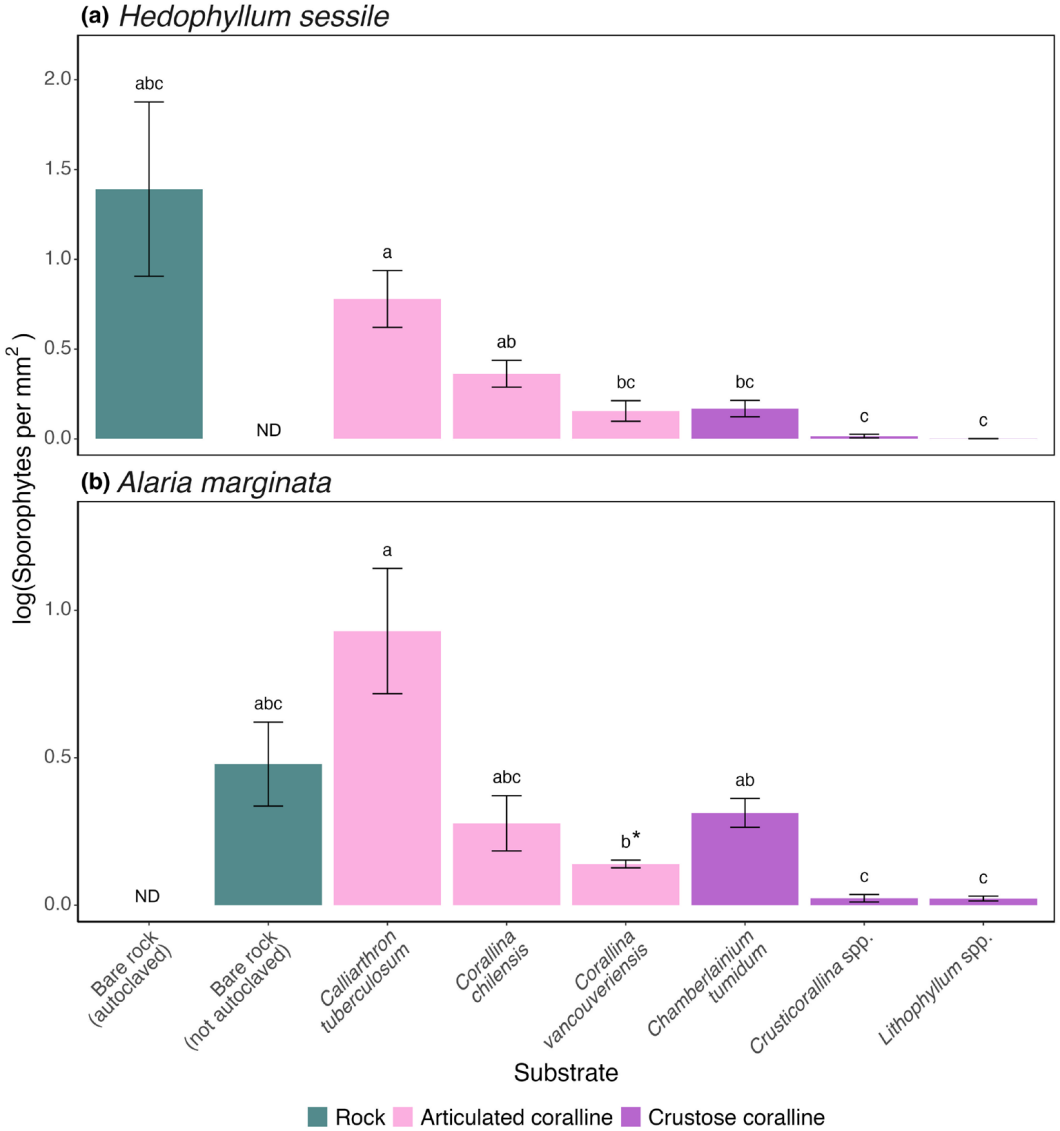
Average sporophyte density on a log‐transformed scale for two intertidal kelps (a: *Hedophyllum sessile*, b: *Alaria marginata*) on rock, three articulated coralline species, and three crustose coralline species. For each kelp species and each substrate, *n* = 9, except for *A. marginata* recruitment on *Corallina vancouveriensis* which had *n* = 5 due to substrate bleaching. Error bars show standard error and lowercase letters show significance groups (*all *p* < 0.05 except the comparison between *A. marginata* on *Calliarthron tuberculosum* versus *Corallina vancouveriensis*, where *p* = 0.057), as calculated with a Games‐Howell post hoc test (Tables S2 and S3). ND refers to categories where no data were collected.

Sporophyte density significantly differed between substrates for both *Hedophyllum sessile* (Figure 2a; Welch's ANOVA, *F*_(6,21)_ = 11.27, *p* < 0.001) and *Alaria marginata* (Figure 2b; Welch's ANOVA, *F*_(6,21)_ = 17.58, *p* < 0.001). Highest average recruitment of *H. sessile* sporophytes occurred on the autoclaved bare rock, at 9.8 sporophytes · mm^−2^ (±5.2 *SE*); however, recruitment on rock was highly variable from 0.1 to 41.8 sporophytes · mm^−2^. Consequently, *H. sessile* sporophyte density on bare rock was not significantly different from any other substrate (Figure 2a; Table S3). *Hedophyllum sessile* recruitment was lowest on *Lithophyllum* spp. (0.002 sporophytes · mm^−2^ ± 0.001 SE) and *Crusticorallina* spp. (0.02 sporophytes · mm^−2^ ± 0.01 *SE*). Sporophyte density of *H. sessile* on these crusts was significantly lower than on the articulated corallines *Calliarthron tuberculosum* and *Corallina chilensis* (Table S3). Interestingly, *H. sessile* recruitment on *Corallina vancouveriensis* was not significantly different from that on any crust (Figure 2a; Table S3). *Calliarthron tuberculosum* was the only articulated coralline with significantly higher *H. sessile* recruitment than the crust *Chamberlainium tumidum* (Figure 2a; Table S3). There was variation in *H. sessile* recruitment among the articulated coralline species, with the highest sporophyte density on *Calliarthron tuberculosum* (1.4 sporophytes · mm^−^^2^ ± 0.4 *SE*) and lowest density on *Corallina vancouveriensis* (0.2 sporophytes · mm^−2^ ± 0.1 *SE*). Recruitment density of *H. sessile* on *Calliarthron tuberculosum* and *Corallina vancouveriensis* was significantly different (Figure 2a; Table S3).

Similar to *Hedophyllum sessile*, the lowest *Alaria marginata* recruitment occurred on the coralline crusts *Lithophyllum* spp. and *Crusticorallina* spp., with a sporophyte density of 0.02 sporophytes · mm^−2^ (±0.01 *SE*) and 0.03 sporophytes · mm^−2^ (±0.01 *SE*), respectively. *Alaria marginata* densities on these two crusts were significantly lower than those on the crust *Chamberlainium tumidum* or on the articulated corallines *Corallina vancouveriensis* and *Calliarthron tuberculosum* (Figure 2b; Table S4). Sporophyte density of *A. marginata* was highest on *Calliarthron tuberculosum* (2.1 sporophytes · mm^−2^ ± 0.7 *SE*). Of the articulated corallines, *Corallina vancouveriensis* had the lowest *A. marginata* recruit density (0.2 sporophytes · mm^−2^ ± 0.02 *SE*), which was nearly significantly different from recruitment on *Calliarthron tuberculosum* (Figure 2b; Games‐Howell test, *t*_(8.06)_ = 3.71, *p* = 0.057). Additionally, *A. marginata* recruitment on any articulated coralline species was not significantly different from the recruitment on *Chamberlainium tumidum* (Figure 2b; Table S4). Recruitment of *A. marginata* on bare rock was not significantly different from any coralline substrates (Figure 2b; Table S4).

**FIGURE 3:**
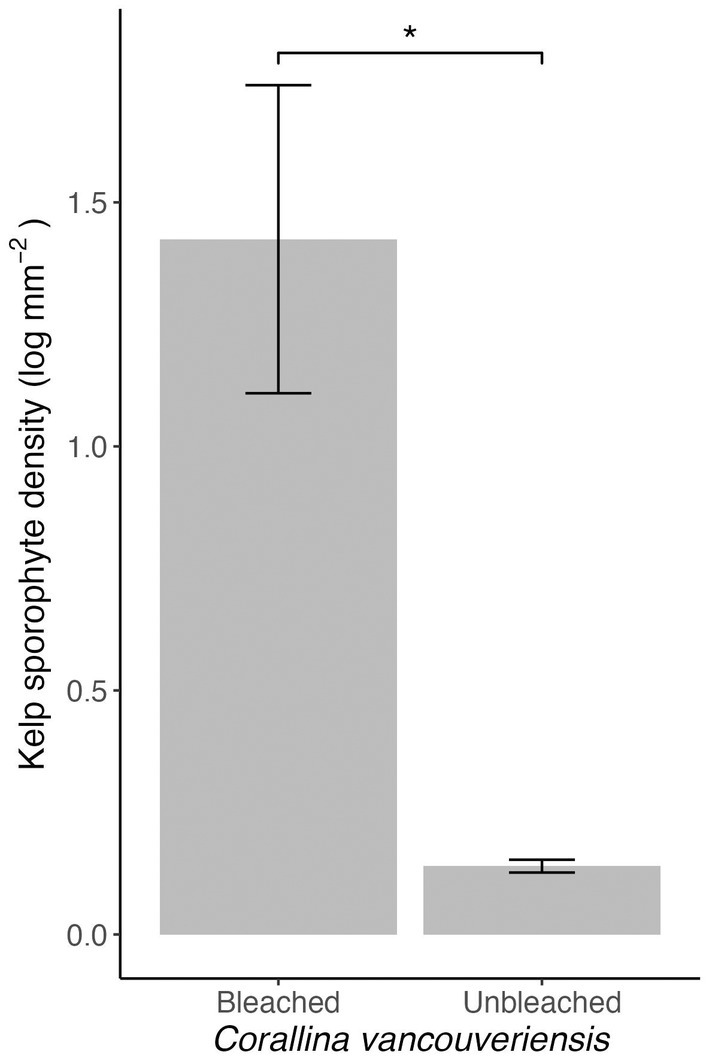
Average log‐transformed *Alaria marginata* sporophyte density on bleached compared to unbleached *Corallina vancouveriensis*. Bleaching occurred during the 7‐week experimental growth period. Error bars show standard error. *n*_Bleached_ = 4, *n*_Unbleached_ = 5, * = *p* < 0.05.

Two weeks after the settlement of *A. marginata* spores, four of the nine *Corallina vancouveriensis* fragments fully bleached. Bleached *Corallina vancouveriensis* were removed from the larger analysis due to a significant change in substrate quality. When compared to unbleached fragments, *A. marginata* sporophyte density was 25 times higher on bleached fragments (Figure 3; Welch's *t*‐test, *t*_(3.01)_ = −4.07, *p* = 0.027).

## DISCUSSION

Using controlled laboratory experiments, we observed that the two intertidal kelps *Hedophyllum sessile* and *Alaria marginata* generally recruited more to articulated corallines than to crustose corallines, reflecting previously observed patterns in the field. However, there was no significant difference between articulated corallines and bare rock, suggesting that extrinsic factors play a large role in decreasing kelp density on rock. Recruitment differed among articulated and crustose species and did not match our hypothesis that *Corallina vancouveriensis* would support the highest kelp recruitment. Importantly, recruitment was higher on bleached than on alive *Corallina vancouveriensis*, suggesting that this articulated coralline species actively deters—rather than induces—kelp recruitment. Our results suggest that the ability to limit kelp epiphytism differs between coralline algal species, which may help explain kelp distribution patterns in the intertidal zone.

Recruitment of both *Hedophyllum sessile* and *Alaria marginata* was not significantly different between corallines and bare rock, which is contrary to field observations of higher kelp density on articulated turfs than rock (McConnico & Foster, 2005; Milligan & DeWreede, 2000) but similar to lab results comparing subtidal kelp recruitment on coralline crusts versus rock (Okamoto et al., 2013). Because the rock in the *H. sessile* trial was autoclaved while that in the *A. marginata* trial was not, factors controlling recruitment on rock in the two trials were likely different. *Hedophyllum sessile* recruitment on autoclaved rock was highly variable. As the autoclave removed any biotic interactions on the rock, differences in recruitment likely reflect physical differences in rock composition and texture. Some rock fragments had large veins of quartz while others were more homogenous and granular (Figure S2). Kelp spore settlement can be influenced by substrate rugosity, surface energy, and hardness, all of which vary with mineral composition (Fletcher & Callow, 1992; Muth, 2012). As such, differences in rock composition between replicates likely increased variation in *H. sessile* recruitment and obscured possible differences in sporophyte density between rock and other substrates. In comparison, the intermediate density of *A. marginata* recruits on rock could have reflected negative interactions with microbes or other algae observed overgrowing the rock, both of which can influence spore and gametophyte survival (Morris, 2018; Reed, 1990). That kelp recruit density was not different between rock and any coralline species in these trials suggests that extrinsic, post‐settlement factors such as herbivory or desiccation likely explain field observations of low kelp density on rock.

**FIGURE 4:**
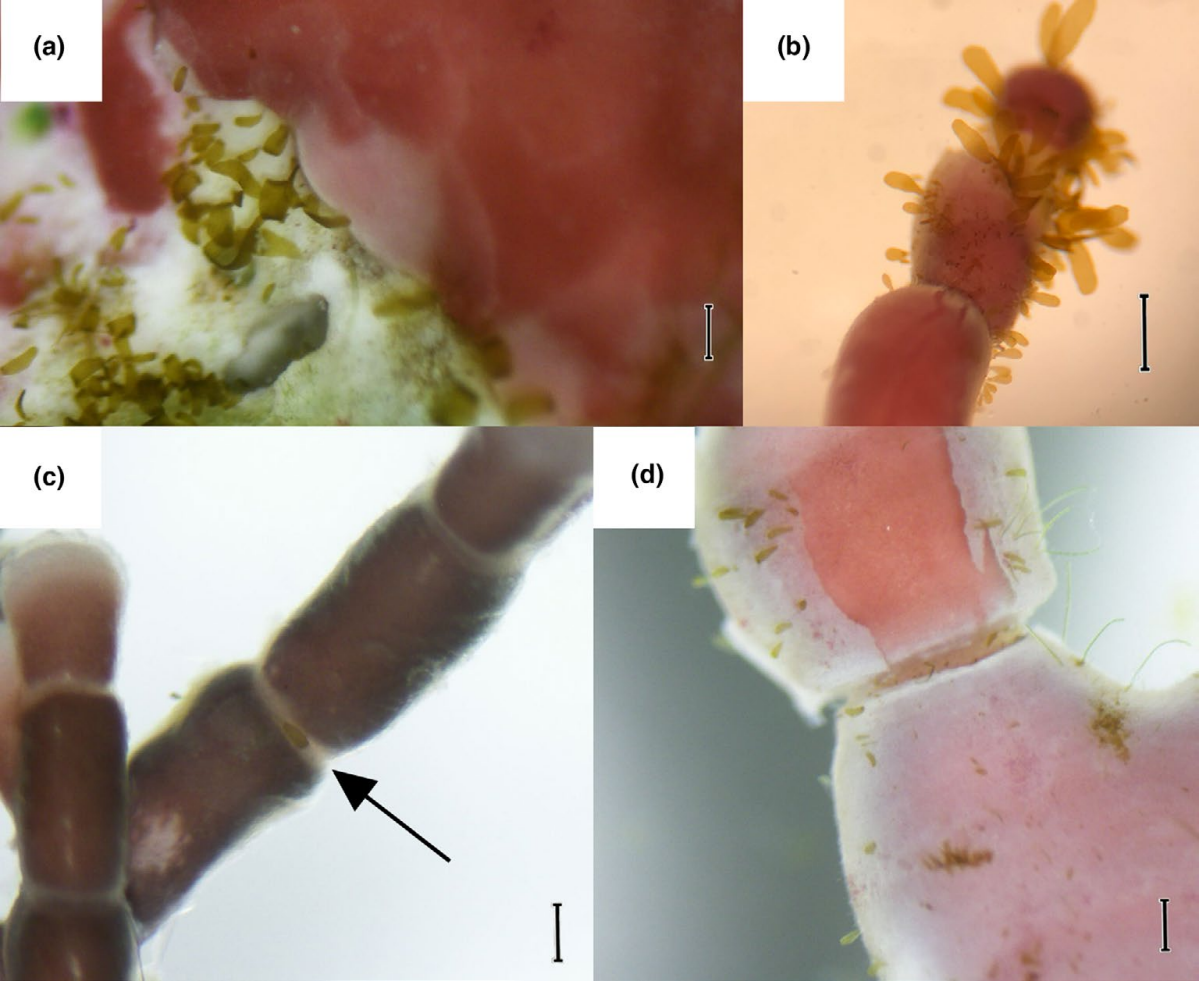
Example photos of kelp sporophyte recruits on various coralline substrates. (a) A cluster of recruits on the bleached area of *Crusticorallina* sp. Scale bar 200 μm. (b) A cluster of recruits on the tip of *Calliarthron tuberculosum*. Scale bar 400 μm. (c) One kelp recruit in a geniculum of *Corallina vancouveriensis*. Scale bar 200 μm. (d) Recruits on the dead surface layer of *Calliarthron tuberculosum*, with a clean area where shedding recently occurred. Scale bar 200 μm.

Kelp recruitment on coralline algae is likely greatly reduced by epiphyte defense mechanisms. Our study provides evidence for this both from quantitative observations of high *Alaria marginata* recruitment on bleached *Corallina vancouveriensis* and qualitative observations of clusters of sporophytes in bleached regions of coralline crusts (Figure 4a). This supports the results of Parada et al. (2017), who observed that bleached coralline crusts supported higher kelp recruitment than live crusts, and contrasts the results of Twist et al. (2023), who observed no influence of crustose coralline bleaching on subtidal kelp recruitment. Our results point to the importance of active, biological defense mechanisms in corallines for suppressing kelp recruitment. These defense mechanisms are likely epithelial shedding and allelopathy, which are driven by cell growth and secondary metabolite production, respectively (Masaki et al., 1984; Keats et al., 1997; Luyen et al., 2009). Coralline bleaching in this experiment occurred after spore settlement, meaning that kelp spores survived the active defense mechanisms and the decreased sporophyte density on live *Corallina vancouveriensis* was mostly due to subsequent gametophyte or juvenile sporophyte death.

Differences in the type and intensity of active defense mechanisms likely explain the differences in kelp recruitment between coralline species. Increased recruitment on articulated corallines compared to crustose corallines may be due to the presence of branch tips and genicula (i.e., uncalcified joints). Sporophyte recruits were observed both on the tips and genicula of articulated corallines in this study (Figure 4b,c) and have previously been observed growing from genicula in the field (Parada et al., 2017). These structures, observed in articulated corallines but not crustose corallines, may serve as refugia from shedding: Branch tips have been observed to shed their epithallia less often than the rest of the thallus (Fujita & Masaki, 1986), and mature coralline genicula may not shed at all because they lack epithelial cells and do not exhibit outward cell division (Johansen, 1981; Martone, 2007). Further, *Calliarthron tuberculosum* and *Chamberlainium tumidum* had the highest kelp recruitment for their morphological groups and were the only corallines observed to develop large flakes of dead tissue over the course of the experiment (Figure 4d). This suggests that *Calliarthron tuberculosum* and *Chamberlainium tumidum* shed their epithallium in large flakes and may require external factors such as wave exposure to effectively remove sporophytes. Kelp recruitment differences between coralline species may also be due to microbiome differences (Lemay et al., 2021; Morris, 2018; Quinlan et al., 2019), although culturing conditions and cleaning protocols prior to the experiment could have changed the microbial composition from those in the field; additional experiments using newly collected corallines and antibiotics should investigate this further.

The general trend of higher kelp recruitment on articulated corallines compared to crustose corallines has ecological implications in terms of community structure. For example, crustose corallines are known to survive in areas with high herbivory while kelps and articulated corallines are more susceptible (Dethier, 1994; Padilla, 1984; Steneck & Dethier, 1994). Our results suggest that even without persistent herbivore pressure, intertidal kelps may not easily return to areas dominated by crustose corallines until articulated corallines return first. However, as crustose corallines do not completely prevent kelp recruitment, it is possible that the sparsely recruited juveniles could still develop into adult kelps at similar densities to other substrates due to the strong self‐thinning that occurs during normal kelp development (Okamoto et al., 2013). Additionally, the species‐specificity of kelp recruitment on articulated corallines might impact intertidal kelp distribution, as stress tolerance and zonation vary between coralline species (Guenther et al., 2022; Guenther & Martone, 2014; Padilla, 1984).

Contrary to our predictions, sporophyte density was higher on *Calliarthron tuberculosum* than on *Corallina vancouveriensis* (Figure 2). Beyond potential differences in epithallial shedding, this recruitment difference may also be due to the larger branch tips of *Calliarthron tuberculosum* providing more room for recruits than *Corallina vancouveriensis*. Importantly, intertidal *Calliarthron tuberculosum* is typically restricted to tide pools where neither *Hedophyllum sessile* nor *Alaria marginata* tend to grow, while *Corallina vancouveriensis* grows alongside intertidal kelps (Barner et al., 2016; Markel, 1996; Padilla, 1984). The higher occurrence of *H. sessile* on *Corallina vancouveriensis* in the field suggests that the recruits that do manage to survive on *Corallina vancouveriensis* may be more likely to reach maturity than recruits on other available substrates, including *Calliarthron tuberculosum*. This increased survival could be explained by factors not included in this experiment, such as water motion aiding in *Calliarthron tuberculosum* shedding as well as *Corallina vancouveriensis* providing protection from herbivory, wave action, and desiccation, which should all be investigated further.

## CONCLUSIONS

Our study showed that there are considerable differences in intertidal kelp recruitment between coralline species in lab conditions, with the highest recruitment on the articulated coralline *Calliarthron tuberculosum* and the lowest recruitment on the coralline crusts *Crusticorallina* spp. and *Lithophyllum* spp. There was a general trend of higher kelp recruitment on articulated corallines than on crustose corallines. Together, this suggests that active epiphyte defense mechanisms greatly reduce kelp recruitment and that these mechanisms are often more effective in some crustose corallines than in articulated corallines. Bleaching facilitated kelp recruitment on *Corallina vancouveriensis*, which opposes the idea that articulated corallines innately increase kelp recruitment. Importantly, *Hedophyllum sessile* had relatively low recruitment on *Corallina vancouveriensis* despite being commonly observed on that coralline substrate in the field, suggesting that external factors create the observed distribution patterns. Similarly, as there were no differences between recruitment on rock versus corallines, other post‐recruitment extrinsic factors likely explain the higher abundance of juvenile kelp on articulated corallines. Future studies should investigate the importance of herbivory, wave action, and desiccation on the recruitment and survival of kelp juveniles on articulated corallines.

## AUTHOR CONTRIBUTIONS

**Ruby Burns:** Investigation (lead); methodology (equal); visualization (lead); writing – original draft (lead); writing – review and editing (equal). **Brenton A. Twist:** Conceptualization (equal); methodology (equal); supervision (equal); writing – review and editing (equal). **Patrick T. Martone:** Conceptualization (equal); funding acquisition (lead); methodology (equal); supervision (equal); writing – review and editing (equal).

## Supporting information


**Figure S1.** The collection sites in BC for algae used in this experiment, indicated by blue pins. The inset shows the relative positions of Clover Point and Ogden Point within Victoria. Corallines were collected at all sites (see Table S1 for details), while *Hedophyllum sessile* was collected at Clover Point and *Alaria marginata* was collected at North Beach.
**Figure S2.** Two replicates of the autoclaved bare rock in the *Hedophyllum sessile* trials with visibly different compositions. (a) A fragment with high sporophyte density. (b) A fragment with no visible sporophytes.
**Table S1.** The locations and dates of the collection of each substrate used for the settlement experiments. All collection sites are within British Columbia, Canada.
**Table S2.** Molecular identification of a subsection of coralline substrates using the rbcL or *psb*A genes.
**Table S3.** Statistical results of a Games‐Howell post hoc test for the comparisons of log‐transformed sporophyte densities of *Hedophyllum sessile* on different substrates.
**Table S4.** Statistical results of a Games‐Howell post hoc test for the comparisons of log‐transformed sporophyte densities of *Alaria marginata* on different substrates.

## References

[jpy70024-bib-0001] Barner, A. K. , Hacker, S. D. , Menge, B. A. , & Nielsen, K. J. (2016). The complex net effect of reciprocal interactions and recruitment facilitation maintains an intertidal kelp community. Journal of Ecology, 104(1), 33–43.

[jpy70024-bib-0002] Burnaford, J. L. (2004). Habitat modification and refuge from sublethal stress drive a marine plant–herbivore association. Ecology, 85(10), 2837–2849. 10.1890/03-0113

[jpy70024-bib-0003] Daume, S. , Brand‐Gardner, S. , & Woelkerling, W. J. (1999). Settlement of abalone larvae (*Haliotis laevigata* Donovan) in response to non‐geniculate coralline red algae (Corallinales, Rhodophyta). Journal of Experimental Marine Biology and Ecology, 234, 125–143.

[jpy70024-bib-0004] Dethier, M. N. (1994). The ecology of intertidal algal crusts: Variation within a functional group. Journal of Experimental Marine Biology and Ecology, 177(1), 37–71. 10.1016/0022-0981(94)90143-0

[jpy70024-bib-0005] Figueiredo, M. A. , De, O. , Kain, J. M. , & Norton, T. A. (2000). Responses of crustose corallines to epiphyte and canopy cover. Journal of Phycology, 36, 17–24. 10.1046/j.1529-8817.2000.98208.x

[jpy70024-bib-0006] Flavin, K. , Flavin, N. , & Flahive, B. (2013). Kelp farming manual: A guide to the processes, techniques, and equipment to farming kelp in New England waters. Ocean Approved.

[jpy70024-bib-0007] Fletcher, R. L. , & Callow, M. E. (1992). The settlement, attachment and establishment of marine algal spores. British Phycological Journal, 27(3), 303–329. 10.1080/00071619200650281

[jpy70024-bib-0008] Fujita, D. , & Masaki, T. (1986). The antifouling by shedding of Epithallium in articulated coralline algae. Marine Fouling, 6(1), 1–5. 10.4282/sosj1979.6.1

[jpy70024-bib-0009] Guenther, R. , & Martone, P. T. (2014). Physiological performance of intertidal coralline algae during a simulated tidal cycle. Journal of Phycology, 50, 310–321. 10.1111/jpy.12161 26988188

[jpy70024-bib-0010] Guenther, R. , Porcher, E. M. A. , Carrington, E. , & Martone, P. T. (2022). Effects of temperature and pH on the growth, calcification, and biomechanics of two species of articulated coralline algae. Marine Ecology Progress Series, 700, 79–93. 10.3354/meps14166

[jpy70024-bib-0011] Guillard, R. R. L. (1975). Culture of phytoplankton for feeding marine invertebrates. In W. L. Smith & M. H. Chanley (Eds.), Culture of marine invertebrate animals (pp. 29–60). Springer. 10.1007/978-1-4615-8714-9_3

[jpy70024-bib-0012] Irving, A. D. , Connell, S. D. , & Elsdon, T. S. (2004). Effects of kelp canopies on bleaching and photosynthetic activity of encrusting coralline algae. Journal of Experimental Marine Biology and Ecology, 310, 1–12. 10.1016/j.jembe.2004.03.020

[jpy70024-bib-0013] Johansen, H. W. (1981). Coralline algae: A first synthesis. CRC Press.

[jpy70024-bib-0015] Keats, D. W. , Knight, M. A. , & Pueschel, C. M. (1997). Antifouling effects of epithallial shedding in three crustose coralline algae (Rhodophyta, Coralinales) on a coral reef. Journal of Experimental Marine Biology and Ecology, 213(2), 281–293. 10.1016/S0022-0981(96)02771-2

[jpy70024-bib-0016] Kelaher, B. P. , Chapman, M. G. , & Underwood, A. J. (2001). Spatial patterns of diverse macrofaunal assemblages in coralline turf and their associations with environmental variables. Journal of the Marine Biological Association of the United Kingdom, 81(6), 917–930. 10.1017/S0025315401004842

[jpy70024-bib-0018] Lemay, M. A. , Chen, M. Y. , Mazel, F. , Hind, K. R. , Starko, S. , Keeling, P. J. , Martone, P. T. , & Parfrey, L. W. (2021). Morphological complexity affects the diversity of marine microbiomes. The ISME Journal, 15(5), 1372–1386. 10.1038/s41396-020-00856-z 33349654 PMC8115056

[jpy70024-bib-0019] Lilley, S. A. , & Schiel, D. R. (2006). Community effects following the deletion of a habitat‐forming alga from rocky marine shores. Oecologia, 148(4), 672–681. 10.1007/s00442-006-0411-6 16598502

[jpy70024-bib-0020] Luyen, Q. H. , Cho, J. Y. , Choi, J. S. , Kang, J. Y. , Park, N. G. , & Hong, Y. K. (2009). Isolation of algal spore lytic C17 fatty acid from the crustose coralline seaweed *Lithophyllum* *yessoense* . Journal of Applied Phycology, 21(4), 423–427. 10.1007/s10811-008-9387-4

[jpy70024-bib-0021] Markel, R. (1996). *Mechanisms underlying the effects of marine herbivores: Implications for a low intertidal kelp community*. [Master's Thesis, University of British Columbia]. UBC Theses and Dissertations. 10.14288/1.0087577

[jpy70024-bib-0023] Martone, P. T. (2007). Kelp versus coralline: Cellular basis for mechanical strength in the wave‐swept seaweed *Calliarthron* (Corallinaceae, Rhodophyta). Journal of Phycology, 43(5), 882–891. 10.1111/j.1529-8817.2007.00397.x

[jpy70024-bib-0024] Masaki, T. , Fujita, D. , & Hagen, N. T. (1984). The surface ultrastructure and epithallium shedding of crustose coralline algae in an ‘Isoyake’ area of southwestern Hokkaido, Japan. Hydrobiologia, 116(1), 218–223. 10.1007/BF00027669

[jpy70024-bib-0025] McConnico, L. A. , & Foster, M. S. (2005). Population biology of the intertidal kelp, *Alaria* *marginata* Postels and Ruprecht: A non‐fugitive annual. Journal of Experimental Marine Biology and Ecology, 324(1), 61–75. 10.1016/j.jembe.2005.04.006

[jpy70024-bib-0026] McCoy, S. J. , & Kamenos, N. A. (2015). Coralline algae (Rhodophyta) in a changing world: Integrating ecological, physiological, and geochemical responses to global change. Journal of Phycology, 51(1), 6–24. 10.1111/jpy.12262 26986255 PMC4964943

[jpy70024-bib-0027] Milligan, K. L. D. , & DeWreede, R. E. (2000). Variations in holdfast attachment mechanics with developmental stage, substratum‐type, season, and wave exposure for the intertidal kelp species *Hedophyllum* *sessile* (C. Agardh) Setchell. Journal of Experimental Marine Biology and Ecology, 254(2), 189–209. 10.1016/S0022-0981(00)00279-3 11077060

[jpy70024-bib-0028] Morris, M. M. (2018). *Microbes among marine giants: Microbial‐macroalgal interactions in Southern California kelp forests* (Publication No. 10937926) [Doctoral dissertation, San Diego State University]. ProQuest Dissertations & Theses Global.

[jpy70024-bib-0029] Muth, A. F. (2012). Effects of zoospore aggregation and substrate rugosity on kelp recruitment success. Journal of Phycology, 48(6), 1374–1379. 10.1111/j.1529-8817.2012.01211.x 27009989

[jpy70024-bib-0030] Okamoto, D. K. , Stekoll, M. S. , & Eckert, G. L. (2013). Coexistence despite recruitment inhibition of kelps by subtidal algal crusts. Marine Ecology Progress Series, 493, 103–112. 10.3354/meps10505

[jpy70024-bib-0031] Padilla, D. K. (1984). The importance of form: Differences in competitive ability, resistance to consumers and environmental stress in an assemblage of coralline algae. Journal of Experimental Marine Biology and Ecology, 79(2), 105–127. 10.1016/0022-0981(84)90213-2

[jpy70024-bib-0032] Parada, G. M. , Martínez, E. A. , Aguilera, M. A. , Oróstica, M. H. , & Broitman, B. R. (2017). Interactions between kelp spores and encrusting and articulated corallines: Recruitment challenges for *Lessonia* *spicata* . Botanica Marina, 60(6), 619–625. 10.1515/bot-2017-0010

[jpy70024-bib-0033] Quinlan, Z. A. , Ritson‐Williams, R. , Carroll, B. J. , Carlson, C. A. , & Nelson, C. E. (2019). Species‐specific differences in the microbiomes and organic exudates of crustose coralline algae influence bacterioplankton communities. Frontiers in Microbiology, 10, 2397. 10.3389/fmicb.2019.02397 31781048 PMC6857149

[jpy70024-bib-0034] R Core Team . (2017). R: A language and environment for statistical computing. R Foundation for Statistical Computing. https://www.R‐project.org/

[jpy70024-bib-0035] Reed, D. C. (1990). The effects of variable settlement and early competition on patterns of kelp recruitment. Ecology, 71(2), 776–787. 10.2307/1940329

[jpy70024-bib-0036] Reed, D. C. , Neushul, M. , & Ebeling, A. W. (1991). Role of settlement density on gametophyte growth and reproduction in the kelps *Pterygophora* *californica* and *Macrocystis* *p* *yrifera* (Phaeophyceae). Journal of Phycology, 27(3), 361–366. 10.1111/j.0022-3646.1991.00361.x

[jpy70024-bib-0042] Schiel, D. R. , & Foster, M. S. (2015). The biology and ecology of giant kelp forests. University of California Press. https://muse.jhu.edu/book/41252

[jpy70024-bib-0037] Schindelin, J. , Arganda‐Carreras, I. , Frise, E. , Kaynig, V. , Longair, M. , Pietzsch, T. , Preibisch, S. , Rueden, C. , Saalfeld, S. , Schmid, B. , Tinevez, J.‐Y. , White, D. J. , Hartenstein, V. , Eliceiri, K. , Tomancak, P. , & Cardona, A. (2012). Fiji: An open‐source platform for biological‐image analysis. Nature Methods, 9(7), 676–682. 10.1038/nmeth.2019 22743772 PMC3855844

[jpy70024-bib-0038] Steneck, R. S. , & Dethier, M. N. (1994). A functional group approach to the structure of algal‐dominated communities. Oikos, 69(3), 476–498. 10.2307/3545860

[jpy70024-bib-0039] Steneck, R. S. , Graham, M. H. , Bourque, B. J. , Corbett, D. , Erlandson, J. M. , Estes, J. A. , & Tegner, M. J. (2002). Kelp forest ecosystems: Biodiversity, stability, resilience and future. Environmental Conservation, 29(4), 436–459. 10.1017/S0376892902000322

[jpy70024-bib-0040] Twist, B. A. , Mazel, F. , Zaklan Duff, S. , Lemay, M. A. , Pearce, C. M. , & Martone, P. T. (2023). Kelp and sea urchin settlement mediated by biotic interactions with benthic coralline algal species. Journal of Phycology, 00, 1–17. 10.1111/jpy.13420 38147464

[jpy70024-bib-0041] Vadas, R. L. , Johnson, S. , & Norton, T. A. (1992). Recruitment and mortality of early post‐settlement stages of benthic algae. British Phycological Journal, 27(3), 331–351. 10.1080/00071619200650291

