# Supplementary material for: Recruitment of intertidal kelps Hedophyllum sessile and Alaria marginata (Laminariales) to articulated and crustose coralline algal species
Source: J Phycol. 2025 May 6;61(3):699–707. doi: 10.1111/jpy.70024 (PMC12168097; doi:10.1111/jpy.70024)
Supplement: Supplementary file 1 — Figure S1. The collection sites in BC for algae used in this experiment, indicated by blue pins. The inset shows the relative positions of Clover Point and Ogden Point within Victoria. Corallines were collected at all sites (see Table S1 for details), while Hedophyllum sessile was collected at Clover Point and Alaria marginata was collected at North Beach. Figure S2. Two replicates of the autoclaved bare rock in the Hedophyllum sessile trials with visibly different compositions. (a) A fragment with high sporophyte density. (b) A fragment with no visible sporophytes. Table S1. The locations and dates of the collection of each substrate used for the settlement experiments. All collection sites are within British Columbia, Canada. Table S2. Molecular identification of a subsection of coralline substrates using the rbcL or psbA genes. Table S3. Statistical results of a Games‐Howell post hoc test for the comparisons of log‐transformed sporophyte densities of Hedophyllum sessile on different substrates. Table S4. Statistical results of a Games‐Howell post hoc test for the comparisons of log‐transformed sporophyte densities of Alaria marginata on different substrates. [file JPY-61-699-s001.docx]

**Supporting Material Caption**

Appendix: Supplementary Methods, Figures, and Tables, containing Figures S1–S2 and Tables S1–S4.

**Appendix**

**Molecular Barcoding Methods**

At least three samples of each morphologically identified coralline taxon were preserved in silica gel following the conclusion of the settlement experiment. Preserved samples were cleaned using forceps before being ground into a powder using a mortar and pestle for DNA extraction. Extractions were done at the University of British Columbia following the protocol outlined by Saunders (2008). The *psb*A or *rbc*L genes were amplified for each specimen with PCR (94°C for 4 min, 39 cycles of 94°C for 1 min, 50°C annealing for 30 s, 72°C extension for 1 min, followed by 72°C final extension for 7 min) using the primer pairs for *psb*A of *psb*A-F1 & R2 (Yoon et al. 2002) and primer pairs for *rbc*L. PCR products were then then sequenced by Génome Québec. Sequences were cleaned using Geneious Prime (2022.2.2), then compared to sequences available in the NCBI database using a BLAST search. Results described in Table S2. Coralline morpho-groups with more than one molecularly identified species, or of an undescribed species, were grouped into the identified genus (ie. *Crusticorallina* spp., *Lithophyllum* spp.), while species that matched their morphological identification were retained in the final analysis. *Chamberlainium tumidum* DNA was not successfully amplified, meaning the final identification was based on morphology alone.


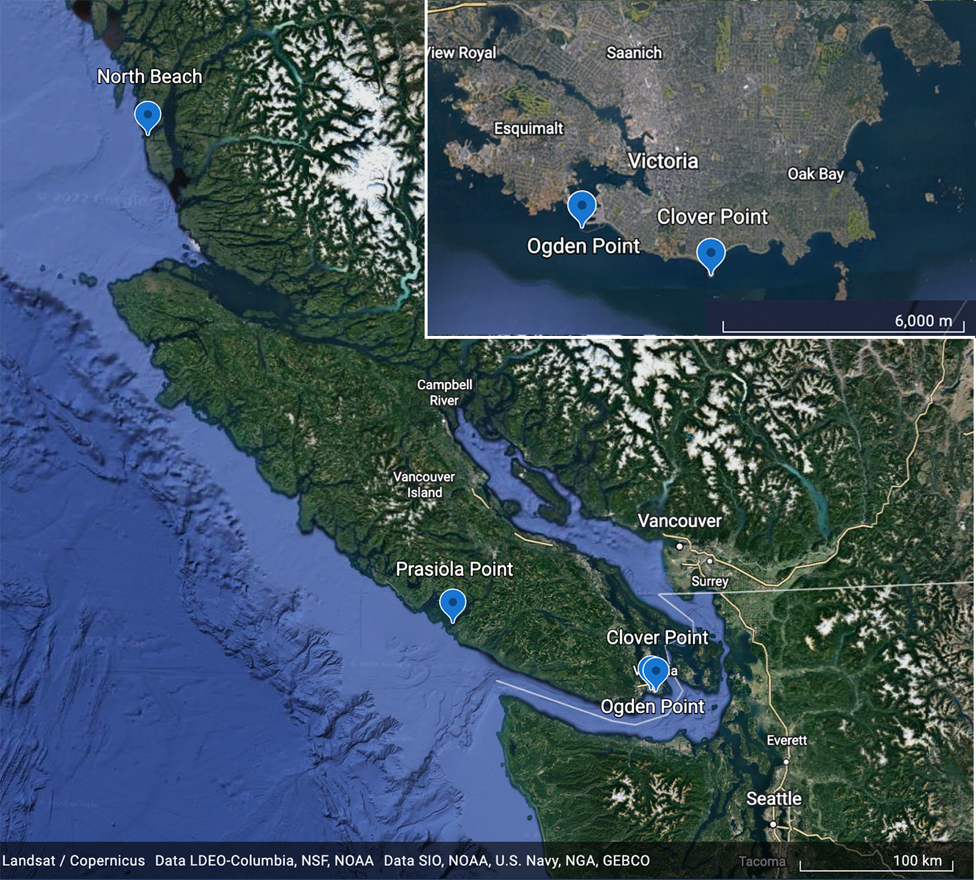


**Figure S1.** The collection sites in BC for algae used in this experiment, indicated by blue pins. The inset shows the relative positions of Clover Point and Ogden Point within Victoria. Corallines were collected at all sites (see Table S1 for details), while *H. sessile* was collected at Clover Point and *A. marginata* was collected at North Beach.


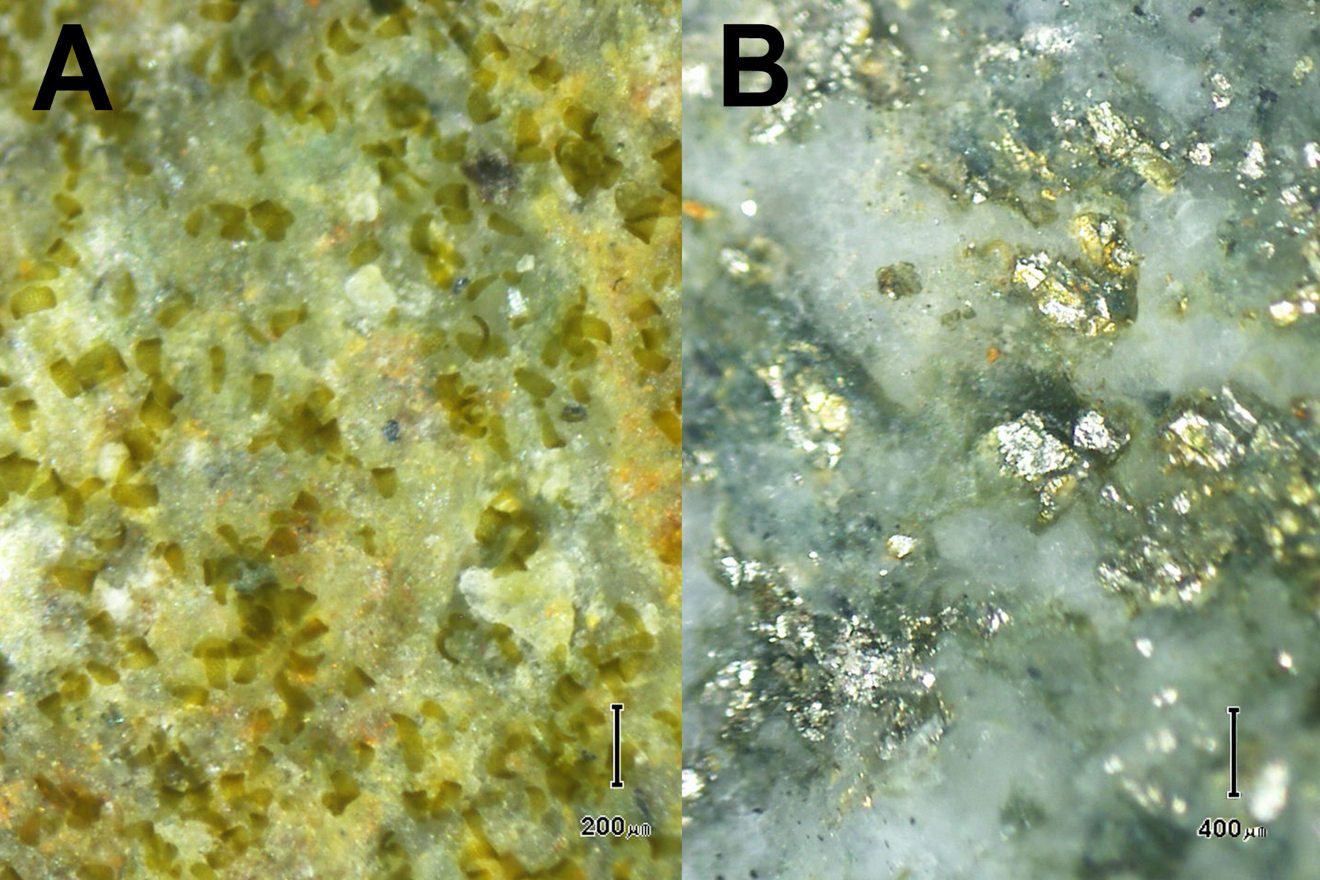


**Figure S2.** Two replicates of the autoclaved bare rock in the *Hedophyllum sessile* trials with visibly different compositions. **A**: A fragment with high sporophyte density. **B**: A fragment with no visible sporophytes.

**Table S1.** The locations and dates of the collection of each substrate used for the settlement experiments. All collection sites are within British Columbia, Canada.

| **Substrate type** | **Species** | **Collection site** | **Coordinates (lat., long.)** | **Collection date** |
| --- | --- | --- | --- | --- |
| Coralline crust | *Crusticorallina*  spp. | North Beach, Calvert Island | (51.6674294, ‑128.1189883) | August, 2021 |
|  | *Lithophyllum*  spp. | North Beach, Calvert Island | (51.6674294, ‑128.1189883) | August, 2021 |
|  | *Chamberlainium tumidum* | Prasiola Point, Bamfield | (48.816658, ‑125.169573) | September 7, 2022 |
| Articulated coralline | *Calliarthron tuberculosum* | Prasiola Point, Bamfield | (48.816658, ‑125.169573) | September 7, 2022 |
|  |  | Ogden Point, Victoria | (48.412939, ‑123.392362) | September 11, 2022 |
|  | *Corallina chilensis* | Prasiola Point, Bamfield | (48.816658, ‑125.169573) | September 7, 2022 |
|  | *Corallina vancouveriensis* | Ogden Point, Victoria | (48.412939, ‑123.392362) | September 11 and October 28, 2022 |
|  |  | Clover Point, Victoria | (48.402695, ‑123.350085) | October 28, 2022 |
| Bare rock | n/a | North Beach, Calvert Island | (51.6674294, ‑128.1189883) | August, 2021 |
|  | n/a | Clover Point, Victoria | (48.402695, ‑123.350085) | October 28, 2022 |

**Table S2.** Molecular identification of a subsection of coralline substrates using the rbcL or psbA genes.

| Initial identification | Gene sequenced | Molecular identification | Identification used in publication | Genbank number |
| --- | --- | --- | --- | --- |
| *Crusticorallina muricata* | psbA | *Crusticorallina muricata* | *Crusticorallina* spp. | PV405021 |
| *Crusticorallina muricata* | psbA | *Crusticorallina painei* | *Crusticorallina* spp*.* | PV405020 |
| *Crusticorallina muricata* | rbcL | *Crusticorallina* sp. | *Crusticorallina* spp*.* | PV405024 |
| *Lithophyllum* sp*.* | psbA | *Lithophyllum* sp. 4 | *Lithophyllum* spp*.* | PV405019 |
| *Lithophyllum* sp*.* | psbA | *Lithophyllum* sp*.* | *Lithophyllum* spp*.* | PV405018 |
| *Corallina chilensis* | psbA | *Corallina chilensis* | *Corallina chilensis* | PV405022 |
| *Corallina chilensis* | rbcL | *Corallina chilensis* | *Corallina chilensis* | PV405026 |
| *Corallina vancouveriensis* | rbcL | *Corallina vancouveriensis* | *Corallina vancouveriensis* | PV405025 |
| *Calliarthron tuberculosum* | psbA | *Calliarthron tuberculosum* | *Calliarthron tuberculosum* | PV405023 |

**Table S3.** Statistical results of a Games-Howell post hoc test for the comparisons of log-transformed sporophyte densities of *Hedophyllum sessile* on different substrates. For significance levels, ns: p > 0.05, *: p < 0.05, **: p < 0.01, and ***: p < 0.001.

| **Group 1** | **Group 2** | **Estimate** | **95% CI (-)** | **95% CI (+)** | **Adjusted *p*** | **Significance level** |
| --- | --- | --- | --- | --- | --- | --- |
| Bare rock | *Calliarthron tuberculosum* | -0.611 | -2.473 | 1.250 | -0.611 | ns |
| Bare rock | *Chamberlainium tumidum* | -1.222 | -3.074 | 0.630 | -1.222 | ns |
| Bare rock | *Corallina chilensis* | -1.028 | -2.880 | 0.824 | -1.028 | ns |
| Bare rock | *Corallina vancouveriensis* | -1.235 | -3.087 | 0.617 | -1.235 | ns |
| Bare rock | *Crusticorallina spp.* | -1.375 | -3.228 | 0.477 | -1.375 | ns |
| Bare rock | *Lithophyllum spp.* | -1.389 | -3.242 | 0.463 | -1.389 | ns |
| *Calliarthron tuberculosum* | *Chamberlainium tumidum* | -0.611 | -1.202 | -0.019 | -0.611 | * |
| *Calliarthron tuberculosum* | *Corallina chilensis* | -0.417 | -1.023 | 0.189 | -0.417 | ns |
| *Calliarthron tuberculosum* | *Corallina vancouveriensis* | -0.624 | -1.219 | -0.029 | -0.624 | * |
| *Calliarthron tuberculosum* | *Crusticorallina spp.* | -0.764 | -1.351 | -0.177 | -0.764 | * |
| *Calliarthron tuberculosum* | *Lithophyllum spp.* | -0.778 | -1.365 | -0.191 | -0.778 | ** |
| *Chamberlainium tumidum* | *Corallina chilensis* | 0.194 | -0.109 | 0.497 | 0.194 | ns |
| *Chamberlainium tumidum* | *Corallina vancouveriensis* | -0.013 | -0.261 | 0.234 | -0.013 | ns |
| *Chamberlainium tumidum* | *Crusticorallina spp.* | -0.153 | -0.341 | 0.035 | -0.153 | ns |
| *Chamberlainium tumidum* | *Lithophyllum spp.* | -0.167 | -0.357 | 0.022 | -0.167 | ns |
| *Corallina chilensis* | *Corallina vancouveriensis* | -0.207 | -0.524 | 0.110 | -0.207 | ns |
| *Corallina chilensis* | *Crusticorallina spp.* | -0.347 | -0.632 | -0.062 | -0.347 | * |
| *Corallina chilensis* | *Lithophyllum sp* | -0.361 | -0.646 | -0.076 | -0.361 | * |
| *Corallina vancouveriensis* | *Crusticorallina spp.* | -0.140 | -0.353 | 0.073 | -0.140 | ns |
| *Corallina vancouveriensis* | *Lithophyllum spp.* | -0.154 | -0.366 | 0.058 | -0.154 | ns |
| *Crusticorallina spp.* | *Lithophyllum spp.* | -0.014 | -0.053 | 0.025 | -0.014 | ns |
| Bare rock | *Calliarthron tuberculosum* | -0.611 | -2.473 | 1.250 | -0.611 | ns |
| Bare rock | *Chamberlainium tumidum* | -1.222 | -3.074 | 0.630 | -1.222 | ns |
| Bare rock | *Corallina chilensis* | -1.028 | -2.880 | 0.824 | -1.028 | ns |
| Bare rock | *Corallina vancouveriensis* | -1.235 | -3.087 | 0.617 | -1.235 | ns |
| Bare rock | *Crusticorallina spp.* | -1.375 | -3.228 | 0.477 | -1.375 | ns |
| Bare rock | *Lithophyllum spp.* | -1.389 | -3.242 | 0.463 | -1.389 | ns |
| *Calliarthron tuberculosum* | *Chamberlainium tumidum* | -0.611 | -1.202 | -0.019 | -0.611 | * |

**Table S4.** Statistical results of a Games-Howell post hoc test for the comparisons of log-transformed sporophyte densities of *Alaria marginata* on different substrates. For significance levels, ns: p > 0.05, *: p < 0.05, **: p < 0.01, and ***: p < 0.001.

| **Group 1** | **Group 2** | **Estimate** | **95% CI (-)** | **95% CI (+)** | **Adjusted *p*** | **Significance level** |
| --- | --- | --- | --- | --- | --- | --- |
| Bare rock | *Calliarthron tuberculosum* | 0.451 | -0.422 | 1.324 | 0.589 | ns |
| Bare rock | *Chamberlainium tumidum* | -0.166 | -0.713 | 0.381 | 0.914 | ns |
| Bare rock | *Corallina chilensis* | -0.201 | -0.784 | 0.382 | 0.891 | ns |
| Bare rock | *Corallina vancouveriensis* | -0.339 | -0.882 | 0.205 | 0.316 | ns |
| Bare rock | *Crusticorallina spp.* | -0.455 | -0.998 | 0.088 | 0.113 | ns |
| Bare rock | *Lithophyllum spp.* | -0.456 | -0.999 | 0.088 | 0.112 | ns |
| *Calliarthron tuberculosum* | *Chamberlainium tumidum* | -0.617 | -1.428 | 0.195 | 0.171 | ns |
| *Calliarthron tuberculosum* | *Corallina chilensis* | -0.652 | -1.477 | 0.174 | 0.159 | ns |
| *Calliarthron tuberculosum* | *Corallina vancouveriensis* | -0.790 | -1.601 | 0.021 | 0.057 | ns |
| *Calliarthron tuberculosum* | *Crusticorallina spp.* | -0.906 | -1.717 | -0.095 | 0.028 | * |
| *Calliarthron tuberculosum* | *Lithophyllum spp.* | -0.907 | -1.718 | -0.096 | 0.028 | * |
| *Chamberlainium tumidum* | *Corallina chilensis* | -0.035 | -0.404 | 0.334 | 1 | ns |
| *Chamberlainium tumidum* | *Corallina vancouveriensis* | -0.173 | -0.360 | 0.014 | 0.074 | ns |
| *Chamberlainium tumidum* | *Crusticorallina spp.* | -0.289 | -0.476 | -0.102 | 0.003 | ** |
| *Chamberlainium tumidum* | *Lithophyllum spp.* | -0.290 | -0.477 | -0.104 | 0.004 | ** |
| *Corallina chilensis* | *Corallina vancouveriensis* | -0.138 | -0.496 | 0.219 | 0.76 | ns |
| *Corallina chilensis* | *Crusticorallina spp.* | -0.254 | -0.612 | 0.103 | 0.212 | ns |
| *Corallina chilensis* | *Lithophyllum spp.* | -0.255 | -0.613 | 0.102 | 0.207 | ns |
| *Corallina vancouveriensis* | *Crusticorallina spp.* | -0.116 | -0.182 | -0.051 | 0.000928 | *** |
| *Corallina vancouveriensis* | *Lithophyllum sp* | -0.117 | -0.178 | -0.056 | 0.001 | *** |
| *Crusticorallina spp.* | *Lithophyllum sp* | -0.000964 | -0.053 | 0.051 | 1 | ns |
| Bare rock | *Calliarthron tuberculosum* | 0.451 | -0.422 | 1.324 | 0.589 | ns |
| Bare rock | *Chamberlainium tumidum* | -0.166 | -0.713 | 0.381 | 0.914 | ns |
| Bare rock | *Corallina chilensis* | -0.201 | -0.784 | 0.382 | 0.891 | ns |
| Bare rock | *Corallina vancouveriensis* | -0.339 | -0.882 | 0.205 | 0.316 | ns |
| Bare rock | *Crusticorallina spp.* | -0.455 | -0.998 | 0.088 | 0.113 | ns |
| Bare rock | *Lithophyllum spp.* | -0.456 | -0.999 | 0.088 | 0.112 | ns |
| *Calliarthron tuberculosum* | *Chamberlainium tumidum* | -0.617 | -1.428 | 0.195 | 0.171 | ns |

**Literature Cited in Supplemental**

Saunders, G.W. (2008). A DNA barcode examination of the red algal family Dumontiaceae in Canadian waters reveals substantial cryptic species diversity. 1. The foliose *Dilsea* – *Neodilsea* complex and *Weeksia*. *Botany*, 86, 773–789.

Yoon, H.S., Hackett, J.D., & Bhattacharya, D. (2002). A single origin of the peridinin-and fucoxanthin-containing plastids in dinoflagellates through tertiary endosymbiosis. *Proceeding of the National Academy of Sciences of the United States of America*, 99, 11724–11729.
